# Increased number of Judo therapy facilities in Japan and changes in their geographical distribution

**DOI:** 10.1186/1472-6963-11-48

**Published:** 2011-02-28

**Authors:** Satoshi Inoue, Mutsuhiro Nakao, Kyoko Nomura, Eiji Yano

**Affiliations:** 1 Department of Hygiene and Public Health, Teikyo University School of Medicine, Tokyo, Japan

## Abstract

**Background:**

Judo therapy is a well established Japanese co-medical profession specializing in outpatient manual treatment of fractures and sprains. Recently, the number of judo therapists has been rapidly increasing as a result of proliferation judo therapy academies. This study examines whether such rapid increases have improved geographical distribution of judo therapy facilities in Japan.

**Methods:**

The number of judo therapy facilities and the population in each municipality were obtained from the Web yellow pages and from Japanese census data for 2004, 2006, and 2008, respectively. Lorenz curves and Gini indices were calculated to demonstrate distributions of judo therapy facilities per 100,000 people. A bootstrapped method was used to identify statistical significances of differences in Gini indices.

**Results:**

In all municipalities, the mean numbers of judo therapy facilities per 100,000 people were 15.3 in 2004, 15.8 in 2006, and 17.6 in 2008. The Gini indices for judo therapy facilities nationally were 0.273 in 2004, 0.264 in 2006, and 0.264 in 2008. The numbers of judo therapy facilities increased significantly between 2006 and 2008 (*p *< 0.05) but the indices did not change significantly in the same period. The Gini indices for local towns and villages remained unchanged and were consistently higher (*p *< 0.05) than those in urban areas throughout the study periods.

**Conclusion:**

Our results suggest that recent increases in the number of judo therapy facilities have not necessarily led to greater equality in their geographic distribution in terms of Gini indices.

## Background

Judo therapy, originating in the Japanese martial art of judo, represents one of Japan's most unique and traditional co-medical professions [1]. Primarily, judo therapists manually treat sprains, bruises, soft tissue damage, fractures, and dislocations in their own offices. Since 1920, judo therapists have required state licensing. Following the 1998 court decision that rejected regulations promulgated by the Japanese Ministry of Health, Labour and Welfare limiting the total number of judo therapists [2], the number of academies training judo therapists increased from 14 to 70. As a result of the increased number of academies, new graduates qualifying as judo therapists began to practice during the period from approximately 2004 to 2006. People have the right for free access to healthcare service under universal coverage of health insurance in Japan; it has been achieved with relatively low cost (340 billion dollars annually for national health expenditure, 9% of GDP). In general, under the Japanese Health Insurance System, judo therapists can operate their facilities independently, and total medical expenditures for judo therapy have been estimated at greater than 3 billion dollars annually [3]. Thus, the practice of judo therapy requires careful monitoring.

Judo therapists are expected to provide complementary and alternative medical treatments, particularly in areas with insufficient medical care services. In this sense, assessing the geographic distribution of judo therapists is important. If the increased number of judo therapists were concentrated primarily in urban areas, which also contain a relatively large number of orthopedists, the overlapping services provided by both professions might lead to conflicts between them. Some orthopedists have rumored that patients at their clinics are recruited by judo therapists. Clearly, for the current increased numbers of judo therapists to provide their services more equitably in terms of geographic distribution would be preferable, but no published studies have examined this possibility.

The Lorenz curve and Gini indices, originally used in economics research to assess income inequality [4,5], were chosen to describe geographic distribution in this study because they can be used to summarize resource distribution on a formal, standardized scale from "0" (even distribution) to "1' (greatest possible unevenness of distribution). Gini indices can provide a standardized basis on which to make judgments on the comparative degrees of geometric unevenness for different manpower resource pools at a point in time, or over a period of time [6,7]. The indices have frequently been used to study disproportionate distributions of health services, including those pertaining to the numbers of physicians and medical facilities [8-13]. For example, using Gini indices, Kobayashi studied the number of physicians in Japanese municipalities and reported the changes in geographical distribution before and after the increase in medical schools [10]. Several reports using Gini indices for the geographical density of physicians, especially practicing physicians and pediatricians, have been published since the mid-1990s [11-13]. To perform the same type of analysis to assess disproportionate distributions of judo therapy facilities, we developed our database of the number of judo therapy facilities in each Japanese municipality and have published two reports on the geographic density of judo therapists [14,15]. However, both studies were cross-sectional in study design and the geographic distribution of judo therapists was not examined over time.

Thus, the objective of this study involved examining recent changes in the geographic distribution of judo therapy facilities in relation to the increase in judo therapy academies in Japan. Assuming that the orthopedists have a primary right to be located where they are, and do not require redistribution themselves, we would like to determine if the expansion of judo therapy in Japan since 1998 has been equitable between urban and rural areas, rather than concentrating in the urban areas where orthopedists have already been established. The hypothesis of this study was that as the number of therapy facilities expanded, so a more equal pattern of provision would be observed. To test this hypothesis, the Gini index of the number of judo therapy facilities at each of the national and regional levels was used as the simple parameter for statistical analysis. Then the Gini indices and their 95% confidence intervals of the number of judo therapy facilities were estimated at two-year intervals during 2004 to 2008, when the number of judo therapy facilities rapidly increased, to compare between 2004 and 2006 and between 2006 and 2008.

## Methods

### Number of judo therapy facilities

The data set, developed from the NTT Internet Townpage Directory of Internet sites [16] in July 2004, 2006, and 2008, was composed of the number of judo therapy facilities in 3,218 municipalities throughout Japan. This method was validated in our previous studies [14,15]; the total number of judo therapy facilities obtained from the Townpage Directory (= 21,995) was close to the number of judo therapy facilities in all prefectures reporting health insurance payments (= 23,199) in 2002. The Internet search used "judo therapist" and the name of each municipality as keywords. Among the search results, only "Sekkotsu-in (facility for bone setting)" and "Seikotsu-in (osteopathy facility)" were selected because other facilities are prohibited from practicing by the Japanese Health Insurance System. This study uses the number of judo therapy facilities instead of the number of judo therapists because no information could be gathered about the latter.

### Population by municipality

The population of each municipality was estimated from census data [17,18], as described in detail in our previous study [15]; the data set can be obtained from the corresponding author upon e-mail request. The incorporated population in July 2008 was used for the analysis of the municipalities consolidated after 2004. The populations of certain major cities were too large for direct comparisons; in these cases, Tokubetsu-ku (specific wards) were used for purposes of comparison. In total, 1,921 municipalities were defined for use in the analysis.

### Analysis

All statistical analyses were performed using the STATA (Ver.9 for Windows) and two-tailed *p *values of less than 0.05 were regarded as statistically significant. Although there were several measurements to report the geographic distributions [10,19], this study used the mean numbers of judo therapy facilities per 100,000 people with their Gini indices according to our previous studies [14,15] and Japanese other studies [6,10]. The Gini indices were calculated based on Lorenz curve, and the procedure of the calculation was as follows. Municipalities were sorted by the number of judo therapy facilities per 100,000 people. Beginning with the municipality with the fewest judo therapy facilities, the x axis of the Lorenz represents the cumulative percentage of the population. The y axis represents the cumulative percentages of judo therapy facilities. Under conditions of complete equality, the cumulative curve would coincide with the 45° diagonal line. Unequal distributions produce cumulative curves below the 45° line. The Gini index is defined as the proportion of the area surrounded by the 45° line and the Lorenz curve in relation to the area below the 45° line; this index ranges from 0 to 1, with higher values indicating larger geographical gaps. In the same way, the Gini indices per 100,000 people were calculated individually for urban areas (= 919) and towns/villages (= 1,002).

The Gini idex is originally a single value reflecting unequal distributions, but the confidence intervals (C.I.s) of the Gini index can be obtained by a bootstrapping procedure [20]. The bootstrap is a computer-intensive method that draws independent samples from the data and calculates the target statistic on each draw. The bootstrap procedure uses the observed data to estimate the theoretical and usually unknown distribution from which the data came [20,21]. Bootstrap samples of the same size as the original sample are repeatedly drawn by sampling with replacement from the observed data. Based on previous studies [22,23] a bootstrap method with 1,000-time randomizations was used to estimate 95% C.I.s of the Gini index. A replacement random sampling was performed 1,921 times from the original database of judo therapy facilities per 100,000 people, and the Gini index was estimated using the created new dataset. This procedure was repeated 1,000 times, and the confidence bands of the Gini index were constructed by multiplicatively expanding the 2.5% and 97.5% points of quantile functions of the simulated data so that the bands have 95% simultaneous coverage over the range of the Gini index. The estimated Gini indices were compared between urban areas and towns/villages for each year. Annual differences in the Gini indices were calculated and the bootstrap method was applied to determine statistical significance [22,23].

## Results

Table 1 shows the number of judo therapy facilities per 100,000 people and the estimated Gini index. Although the general population gradually decreased during the period studied, the number of judo therapy facilities consistently increased, resulting in an increase of the number of judo therapy facilities per 100,000 people. The number of judo therapy facilities in the entire country increased by 5.3% in 2006 and 8.3% in 2008 per 100,000 people compared to the values obtained in 2004 and 2006, respectively. This increase was significant only in 2008, which posted the same increase in urban areas. In contrast, no significant changes were found for towns/villages in both 2006 and 2008.

**Table 1:** The number of judo therapy (J.T.) facilities, their distribution per 100,000 people, and Gini indices in all areas, urban areas, and towns/villages in 2004, 2006, and 2008.

	2004	2006	2008
Population (×1000)			
All areas (n = 1,921)	127,902	127,758	126,931
Urban areas (n = 919)	114,557	114,552	114,049
Town/villages (n = 1,002)	13,345	13,206	12,882

Number of J.T. facilities			
All areas	22,774	23,996	25,989
Urban areas	20,888	22,045	23,906
Towns/villages	1,886	1,951	2,083

J.T. facilities per 100,000 population*			
All areas	15.3 ± 11.8 (14.7-15.7)	15.8 ± 12.2 (15.3-16.4)	17.6 ± 14.0 (17.0-18.2)†
Urban areas	18.0 ± 9.8 (17.4-18.6)	19.0 ± 9.9 (18.3-19.6)	20.9 ± 12.1 (20.2-21.7)†
Towns/villages	12.8 ± 12.9 (12.0-13.6)	13.1 ± 13.3 (12.2-13.9)	14.8 ± 14.9 (13.6-15.4)

Mean Gini (95% C.I.)*			
All areas	0.273 (0.261-286)	0.264 0.253-0.276)†	0.264 (0.251-0.276)
Urban areas	0.258.248-0.267)	0.247 0.238-0.257)†	0.248 (0.239-0.257)
Towns/villages	0.407 (0.394-0.419)	0.404 (0.392-0.417)	0.400(0.388-0.413)

Mean values ± standard deviations (95% confidence intervals, C.I.s) of J.T. facilities per 100,000 people are presented; the mean Gini indices (95% C.I.s) of their distributions were estimated using the bootstrap method.

^† ^The differences in values were significant (*p *< 0.05, two-tailed t-test) when compared to those in the previous 2 years.

**Figure 1:**
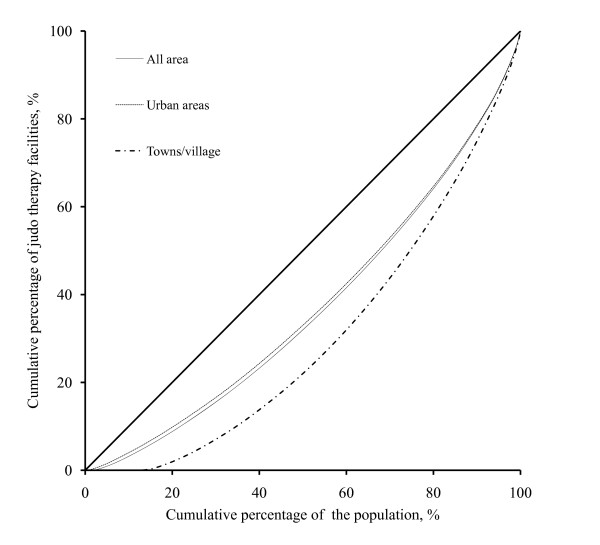
**Lorenz curves of distributions of judo therapy facilities in all areas, urban areas, and towns/villages in 2008**. The 45° diagonal line represents a completely even distribution. Unequal distributions produce cumulative curves below the 45° line.

Compared to the values in the previous 2 years, the degrees by which the Gini index changed (95% C.I.) were -0.009 (-0.013, -0.006) in 2006 and 0.0005 (-0.006, 0.007) in 2008 in all areas; they were -0.010 (-0.013, -0.007) in 2006 and 0.0006 (-0.005, 0.006) in 2008 in urban areas, and -0.003 (-0.009, 0.003) in 2006 and -0.004 (-0.011, 0.003) in 2008 in towns/villages. This indicates that the Gini index significantly decreased in 2006 but not in 2008 in all areas and in urban areas but that it did not decrease significantly in either 2006 or in 2008 in towns/villages The Gini index was statistically higher (all *p *< 0.05) in urban areas than in towns/villages in 2004, 2006, and 2008. The Lorenz curves of judo therapy facilities per 100,000 people in 2008 are presented for all, urban, and town/village areas in Figure 1.

## Discussion

The present study demonstrates that the density of judo therapy facilities per population unit has increased, irrespective of area, from 2004 to 2008 along with the increase in the absolute number of judo therapy facilities. In particular, the extent to which judo therapy facilities per population unit increased in 2008 was statistically significant and higher than that in 2006 by a factor of more than 1.5. However, this increase in 2008 did not reflect equality in the geographic distribution of judo therapy facilities according to the Gini index. Rather, it reflects a trend toward increased geographical gaps between urban areas and the rest of Japan in 2008.

These findings are important because under the current laws and regulations governing medical delivery systems, the Japanese government cannot intervene in choices about where medical practitioners practice. For example, Japan's physician manpower policy during the 1970s involved increasing the number of medical students and medical schools from 65 to 79 to address the shortage and maldistribution of physicians resulting in communities without doctors. The number of newly certificated physicians increased from approximately 4,000 to 8,000 per year by the mid-1980s. However, the inequality in physician distribution did not improve between 1980 and 1990 according to a previous Japanese study [10], suggesting that simply increasing the supply of medical providers does not constitute an advisable health policy. Rather, a policy that alleviates the maldistribution of medical providers should be developed. In the present study, the Gini indices significantly decreased in all areas and in urban areas only in 2006, even though the extent to which judo therapy facilities per population unit increased during this year was lower than that during 2008. Because the number of judo therapy facilities per population unit has remained at greater than 20 per 100,000 in urban areas since 2008, it seems clear that the rapid and substantial growth in the number of qualified judo therapists might not naturally match the geographic distribution of the need for judo therapy facilities.

In the present study, national data were not used for two reasons, even though all judo therapists are required to report to the designated public health center. First, the statistics on judo therapists are organized according to each public health center but not according to each municipality [24]. Second, these data were not completely accurate insofar as they might have included defunct businesses because reporting closures of these facilities is not required [14,15]. We finally decided to gather information about judo therapy facilities from the Townpage of each municipality, and the number obtained via this method was within 5% of the number of judo therapy facilities in all prefectures reporting health insurance payments. The number of facilities may represent a good surrogate for the number of therapists because in most cases, each facility contains only one practicing judo therapist. Using registration data obtained from the Japanese Judo Therapists' Association [25], we estimated that an average of 1.09 therapists worked in each facility during the period studied.

Before making remarks, several limitations should be noted. First of all, this study is not an analysis of the economics of service provision, but a use of a particular descriptive technology in assessing distributions of judo therapy facilities. Spatial inequality matters are largely due to the time price associated with any health facility, as well as other economic phenomenon including spatial access to health facilities [26,27]. Because geographical distribution of medical service provider directly relates to urgent need of human life in the community, distribution per se should be analyzed apart from economics. Financial aspects of Judo therapy facilities are important but beyond our scope requiring totally different data set and method for analysis. Thus the results of time-changes in geographic distribution of judo therapy facilities were simply shown in this study. In the future a variety of economic factors need to be considered to interpret our results before approving or opposing health-care policy for the number of judo therapy facilities. Second, this study is limited by its use of a municipality-based method to determine the number of judo therapy facilities and residents; the scale and nature of the facilities could not be assessed. These were grouped data, and the possible effects of 'ecological fallacy' should also be firmly considered for the interpretation of the results [28]. Also, the number of judo therapy facilities was divided by 100,000 people as the only indicator of 'need'. This was because the national data of health-care facilities have usually been published as a unit of per 100,000 residences in Japan, but we should bear in mind that different 'needs' indicators normally produce different inequality estimates: all of which have implications for health policy and planning competing health priorities. Third, the effects of unions of municipalities on the Gini indices should be considered. The Gini index is itself affected by the number of subjects analyzed [17,18], and the indices for small towns and villages are smaller when such towns or villages are combined into larger cities. However, the number of united cities, towns, and villages was limited (= 27) from 2006 to 2008 and does not appear to account for changes in the geographical differences characterizing Japan in 2006. Fourth, four-year study period was relatively short to observe the change of distribution of judo therapy facilities. However, we were specifically interested in the change of distribution of judo therapy facilities from the start of drastic change of graduates qualifying as judo therapists (i.e., the years 2004 to 2006) in this study, and we recognize that the future study should be continued to observe the distribution of judo therapy facilities.

In spite of these limitations, we demonstrated that judo therapy facilities are widely but unevenly distributed. We suggest that recent increases in the number of judo therapy facilities do not necessarily lead to amelioration of inequalities in their geographic distribution. A large portion of elderly individuals live in nonurban areas, and this fact seems to reinforce the need for judo therapy in such areas [29]. Geographic distributions of health services are affected by forces of demand and supply, and in the future we would like to assess the needs for judo therapy and the number of qualified judo therapists comprehensively in all regions before considering intervention plans to motivate judo therapists to practice in the underserved local areas.

## Conclusions

The numbers of judo therapy facilities increased significantly between 2006 and 2008 in Japan, but the Gini indices did not change significantly in the same period. The Gini indices for local towns and villages remained unchanged and were consistently higher than those in urban areas throughout the study periods. These results suggest that recent increases in the number of judo therapy facilities have not necessarily led to greater equality in their geographic distribution in terms of Gini indices.

## Competing interests

The authors declare that they have no competing interests.

## Authors' contributions

SI collected the data, performed statistical analysis, and wrote the manuscript. MN and EY have made substantial contributions to conception and design, and have been involved in drafting and revising the manuscript. KN made substantial contributions to analysis and interpretation of data. All authors read and approved the final manuscript.

## Pre-publication history

The pre-publication history for this paper can be accessed here:

http://www.biomedcentral.com/1472-6963/11/48/prepub
